# Pilot Demonstration of Hot Sheet Metal Forming Using 3D Printed Dies

**DOI:** 10.3390/ma14195695

**Published:** 2021-09-30

**Authors:** Jaume Pujante, Borja González, Eduard Garcia-Llamas

**Affiliations:** 1 Eurecat, Centre Tecnològic de Catalunya, Unit of Metallic and Ceramic Materials, Plaça de la Ciència, 2, 08243 Manresa, Spain; eduard.garcia@eurecat.org; 2 Gestamp, Autotech Engineering Spain SL, Polígono Industrial Ca N’Estella, Carrer Edison, 4, 08635 Sant Esteve Sesrovires, Spain; bogonzalez@gestamp.com

**Keywords:** 3D printing, additive manufacturing, cooling, press hardening, tooling

## Abstract

Since the popularization of press hardening in the early noughties, die and tooling systems have experienced considerable advances, with tool refrigeration as an important focus. However, it is still complicated to obtain homogeneous cooling and avoid hot spot issues in complex geometries. Additive Manufacturing allows designing cavities inside the material volume with little limitation in terms of channel intersection or bore entering and exit points. In this sense, this technology is a natural fit for obtaining surface-conforming cooling channels: an attractive prospect for refrigerated tools. This work describes a pilot experience in 3D-printed press hardening tools, comparing the performance of additive manufactured Maraging steel 1.2709 to conventional wrought hot work tool steel H13 on two different metrics: durability and thermal performance. For the first, wear studies were performed in a controlled pilot plant environment after 800 hot stamping strokes in an omega tool configuration. On the second, a demonstrator tool based on a commercial tool with hot spot issues, was produced by 3D printing including surface-conformal cooling channels. This tool was then used in a pilot press hardening line, in which tool temperature was analyzed and compared to an equivalent tool produced by conventional means. Results show that the Additive Manufacturing technologies can be successfully applied to the production of press hardening dies, particularly in intricate geometries where new cooling channel design strategies offer a solution for hot spots and inhomogeneous thermal loads.

## 1. Introduction

Press hardening, also known as hot stamping, is a non-isothermal forming process for sheet metals, where forming and quenching take place in the same forming step [1]. In direct press hardening, blanks are austenitised at a temperature between 900 and 950 °C for 4–10 min inside a furnace. The austenitised blank is then transferred to a set of cooled dies where it is formed and quenched in a single step, ensuring full martensitic microstructure. The total cycle time including transfer, forming and quenching typically takes 15 to 20 s [2]. 

Use of press hardening allows producing lightweight components with very high mechanical properties and complex shape while avoiding problems, such as spring-back on the component or damage including fracture on the tools, associated with cold forming of Ultra-High Strength Steels (UHSS). For this reason, since the turn of the century press hardening has steadily gained relevance, starting off as a specialist application and turning into a staple technique in the production of safety cage products for the automotive industry [3].

One of the defining traits of press hardening and, at the same time, the main bottleneck for part quality and process efficiency, is the need to provide a successful component quench, and therefore the need for efficient tool refrigeration. Parts need to be quenched above a critical cooling rate of approximately 30 K/s [1] to achieve full martensitic transformation; failing to do so will result in mixed microstructure leading to lower mechanical properties or geometrical distortions. 

Being a hot topic, the issue of refrigeration has been approached in a multitude of forms. While some technologies such as high conductivity steels can be used to lower overall tool temperature and improve cooling efficiency [4], the main working line involves the correct design of tool cooling circuits [5]. 

The most conventional option is to provide refrigeration through a series of machined bores forming closed-loop channels inside the die. The current state of the art provides workable solutions in most cases, but faces limitations with very complex surface geometries, where the channels may fall too far away from the surface leading to insufficient cooling or hot spots. 

One possible solution are core-shell designs [5], where a water chamber is created between an inner block giving structural integrity to the tooling and an outer shell in contact with the component. While offering good cooling performance, this solution is awkward to implement and maintain. A final option is producing cast tools, with in-cast cooling channels. While this allows very flexible design, it is complex to implement, and the catalogue of materials is very limited: a steel composition is required which can be competitively cast and offers the mechanical and functional properties of a hot work tool steel.

As a result, cooling channels bored into a massive tool have remained the industry standard since the mainstream adoption of press hardening, with small improvements such as division of the tool into smaller sections facilitating the boring of refrigeration circuits. This approach is successful in most cases, but still finds its limits in complex geometries, where cooling circuits may fall too far away from the tool surface.

In recent years Additive Manufacturing and 3D printing have experienced considerable improvements both on technical results and on cost competitiveness. Their inclusion in forming tools of tailor-designed cooling solutions has been already explored in detail in the plastic molding industry, where the advantage provided by an increase in cooling efficiency quickly translates into shorter cooling time and therefore higher production rate [6,7]. These technologies were already identified as potential ways to obtain complex cooling circuits in press hardening [5] and significant effort is being dedicated to evaluate their feasibility, with works as early as in 2014 [8]; a comprehensive review was recently (2020) produced by Chantzis et al. [9], identifying the interest of the application despite the lack of formal experimental studies.

One possibility for producing conforming cooling systems on metal forming dies consists in milling channels directly on the tool surface, and then covering them with an overlay generated through Additive Manufacturing technology. This method allows for completely conformal channels and is reminiscent of the conventionally produced core-shell construction sometimes used in press hardening [5]. Cortina et al. explored this approach in work [10]: cooling channels were milled directly on the tool surface and covered by a tool steel overlay using Laser Metal Deposition. After basic trials on a flat die, a complex geometry demonstrator was manufactured, proven to be feasible and evaluated by means of Finite Element Modelling, showing promising results. A similar approach with a more complex implementation was demonstrated by Hong et al. [11], using a concept dubbed HMAM (heterogeneous material additive-manufacturing). In this case, structural steel is used as the base “cooling block”, with channels milled directly on its surface; these channels are then closed by the application of an additive layer of high thermal conductivity steel HTCS150 on top of this base block. Again, promising results were obtained in a truncated B-pillar geometry.

This strategy offers potential for good results combined with a relatively easy implementation and has shown to be technically viable. Its main limitations are, on the one hand, a certain susceptibility to problems related to defects in the AM layer, or to durability issues such as Stress Corrosion Cracking, which can lead to leaks or failure through the welded overlay. Additionally, these techniques do not offer the full advantages of free design through 3D printing.

Complete freedom of design can be obtained by producing the complete tool using a 3D printing technology such as Direct Metal Laser Sintering. In this case, the tool is built from the ground up, offering the possibilities such as generating internal cavities, additional paths for sensors or control systems or tailored entrances for water circuits. This design strategy is equivalent to the plastic molding examples shown before [6,7].

Again, the concept is a natural idea and some works in the published literature already explore its implementation. Chantzis et al. [12] analyzed using Finite Element Modelling the design of 3D printed tools with tailored cooling channels, in this case for hot stamping of aluminum sheet. Their work discusses with the aid of FEM how, through the use of 3D printed channels and the incorporation of lattice structure, the thermal behavior of the tool can be modified, and modelling results are validated in a series of experiments. 

All these references show that the application of full 3D Printing to the manufacture of sheet metal forming dies, and press hardening dies in particular, is identified as an interesting case study. However, despite the interest, the published literature still lacks explicit experiences showcasing such an application [9] demonstrated in an industrially relevant setting, as opposed to FE Modelling analyses, which are supported by abstract laboratory tests.

Regardless of the technique used, one of the issues arising when considering Additive Manufacturing for a metal forming tool is the tool material itself; in this case, the AM technology applied is relevant. Laser Cladding-based techniques such as Direct Laser Deposition can be more permissive in terms of deposited material; examples in the literature include a range, from conventional materials such as hot work steel H13 [10] to specialty tool steels such as the high thermal conductivity HTCS150 [11]. Powder bed technologies, such as Direct Metal Laser Sintering are less forgiving and offer a more limited catalogue of materials; usual choices include Stainless Steel grades [6] or Maraging Steels [6,13], the latter being more suited to press hardening due to the higher hardness achievable. In particular, Vikhareva et al. [13] studied the tribological performance of 3D printed Maraging steel taking into account the typical wear mechanisms in press hardening; results validated that the material could offer performance similar to a conventional hot work tool steel, at least in an abstract laboratory setting.

This work explores the use of 3D printed dies into two industry-relevant press hardening pilot trials in order to bridge the gap existing between laboratory tests and FE Modelling, and industrial applications.

On the one hand, an omega shape tool has been used to confirm the feasibility of building a tool combining 3D printed material and conventional machined tool steel. This test allows evaluating the wear and damage mechanisms appearing in continued production and demonstrates that 3D printed material shows no anomalous performance issues. On the other hand, a second demonstrator has been designed and built to evaluate the improvements in cooling efficiency available through free design of cooling channels. In both cases, Maraging Steel has been used as 3D printing material, and its performance compared to commonly used hot work tool steel H13.

Results show that 3D printing is a technically realistic choice for manufacturing press hardening dies, implying that new research works in the line of developing advanced cooling strategies, or validating new tool materials may have an almost immediate industrial application.

## 2. Materials and Methods

### 2.1. Materials

AM samples were produced using a commercial SLM solution provided by supplier EOS (DMLS, Direct Metal Laser Sintering [14]). The material used, under the commercial denomination MS1, corresponds to a 1.2709 Maraging Steel (Table 1), a special application hot work tool steel hardened by artificial aging of a very low C martensite. Due to their low warping and aging heat treatment, Maraging steels are a common candidate for 3D printing [6,13,15], and 1.2709 is present in the catalog of various manufacturers. All samples were built in the conventional 3D printing parameters, separated from the production base and heat treated by artificial aging for 6 h at 490 °C, reaching a final hardness of 52–54 HRC. Machining was used to generate threads for attaching screws and water manifolds and to obtain the final surface finish.

**Figure 1:**
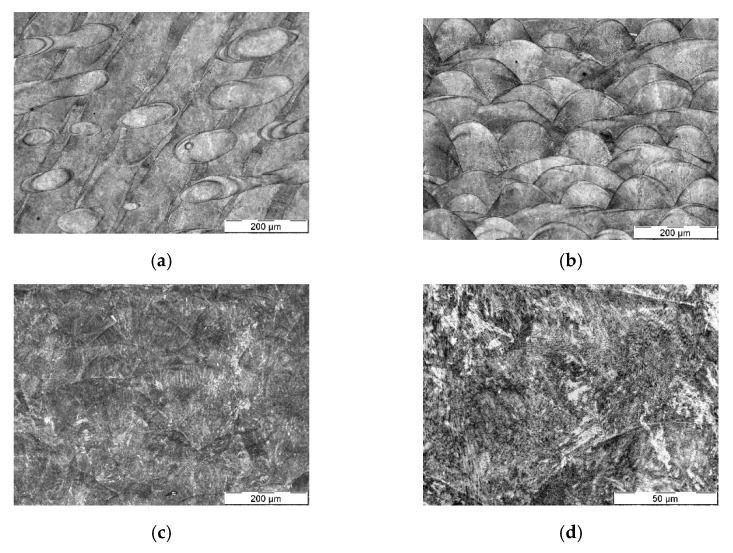

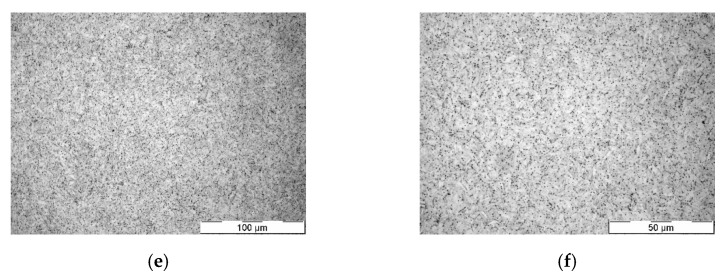
Optical microscopy images showing the microstructure of the studied tool steels; (**a**,**b**) MS1 material as-printed: (**a**) x-y plane and (**b**) y-z plane; (**c**,**d**) x-y plane of MS1 material after aging, showing a fine dispersion of precipitates, original grain can still be intuited; (**e**,**f**) conventionally produced (wrought) QRO90 tool steel, quenched and tempered to 50–52 HRC.

Microstructural analysis of the *as printed* material (Figure 1a,b) shows a morphology reminiscent of a weld overlay in a criss-cross pattern; this is consistent with the accumulation of material during printing and observations in the literature [15]. No significant instances were observed of defects such as porosity, cracks, or segregations. After heat treatment (Figure 1c,d), the grain is obscured by a fine distribution of precipitates; again, this is consistent with observations in the literature [13,15]. The original morphology appears to be unmodified and can still be recognized by careful observation, particularly in low-magnification images (Figure 1c).

As a reference, conventional tool steel grade QRO90 was used, a commercial variation of hot work tool steel H13 (Table 1). This wrought tool steel was soft-machined, heat treated by quenching and tempering to a final nominal hardness of 50–52 HRC and finished. The microstructure obtained (Figure 1e,f) corresponds to the expected structure of a hot work tool steel, consisting in a matrix of tempered martensite with some small, rounded metal carbides, mainly VC. While the hard martensitic matrix strengthened by precipitates formed during tempering, is similar to the maraging matrix, the presence of microscopic carbides supposes a significant difference. The grain morphology shows basically equiaxial grains, formed during the heat treatment of the wrought block of material, as opposed to the microstructure oriented in planes in 3D printed material. 

### 2.2. Pilot Trials in Omega Tool Configuration

The first pilot trial tests were performed in an omega tool configuration, with the aim of detecting any anomalous performance of the material in serial production and comparing the wear behavior of the studied material with that of a reference tool steel. 

#### 2.2.1. Test Configuration

A laboratory scale press hardening line was set up for these trials, aiming to reproduce the main conditions of an industrial environment. Set up of this experimental configuration was discussed in [16], where it was verified that the main wear mechanisms observed corresponded to those in industrial press hardening tools.

Heating is conducted in a 3 m long continuous roller-hearth convection-radiation furnace, working in open (oxygen-containing) atmosphere. Austenitized sheets are manually transferred to 150 t hydraulic press equipped with a set of cooled dies, where hot stamping is performed.

**Figure 2:**
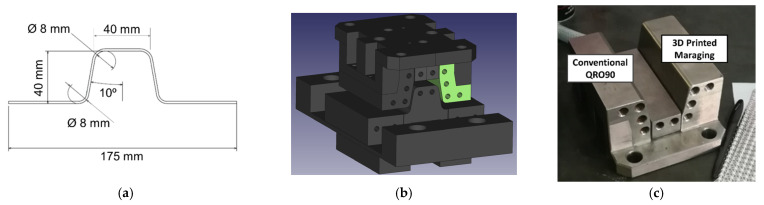
Details about the Omega Tool configuration: (**a**) Component geometry, based on a B-pillar cross-section; (**b**) CAD view of the tool assembly, showing how the die, punch, and blank holder form the component geometry, 3D printed insert is highlighted in green; (**c**) view of the die, formed by two inserts (QRO90 and 3D printed 1.2709 material) mounted on a structural steel base.

Component geometry was based in an omega-shape, a common concept in press hardening literature. The final geometry was defined based on a survey of cross-section measurements obtained from production B-pillars during research project HardTrim (Figure 2a). The dimensions thus defined were considered to combine a relevant example of the working conditions of industrial tooling with ease of inspection. Tool laterals were designed as two symmetrical replaceable inserts. One side was manufactured in commercial hot work tool steel QRO90 heat treated to 50–52 HRC. The other side was produced with steel MS1 through Additive Manufacturing and aged to 52–54 HRC. This allowed direct side-by-side comparison of the two materials (Figure 2b,c).

Using this setup, 130 mm × 250 mm, 1.7 mm thick, commercial USIBOR 1500 AlSi blanks were stamped with cycle time of 35 s, composed of approximately 5 s furnace-die transfer time (manual transfer), 3 s tool closing time at approximately 40 mm/s press velocity, 10 s cooling time and 5 s tool opening time, the rest of the cycle time being extraction of the formed component and idle time. 

Austenitisation was conducted at 930 °C with a total 320 s furnace time. Inlay temperature (blank temperature at closing) was approximately 830 °C. Press closing force used was 70 t, leading to an average closing pressure of 30 MPa over the projected component area.

Tools had cooling channels 8 mm in diameter, situated 10 mm below the tool surface (distance between surface and center of the channel). Water at 16 °C was circulated through these channels at approximately 4.5 L/min, no significant heating of the water was measured at the exit of the channel. Tools were measured to reach 80 °C at their surface after 25 blanks, assumed close to steady state. This range of temperatures is representative for industrial press hardening tools [17].

#### 2.2.2. Analyses

Analyses were performed using Stereo Microscopy for 2D measurements and an Alicona InfiniteFocus SL light profilometer for 3D and topography evaluation. Measurements included adhesion of material on the tool radius, wear in the tool flanks and appearance of rough “combined wear” fringes; comparison of these wear mechanisms to industrial tooling is discussed in [16].

Tools were analyzed at various points of the production: Initial state, 250 cycles, 500 cycles and 800 cycles

### 2.3. Thermal Performance Demonstrator

The aim of these tests was to evaluate if the conformal cooling channels produced by Additive Manufacturing resulted in better cooling performance of the tool.

#### 2.3.1. Demo1 Tooling

The tooling used was a demonstrator tool approximately 150 mm in length corresponding to a section of an industrial tool with complex geometry resulting in poor cooling performance.

**Figure 3:**
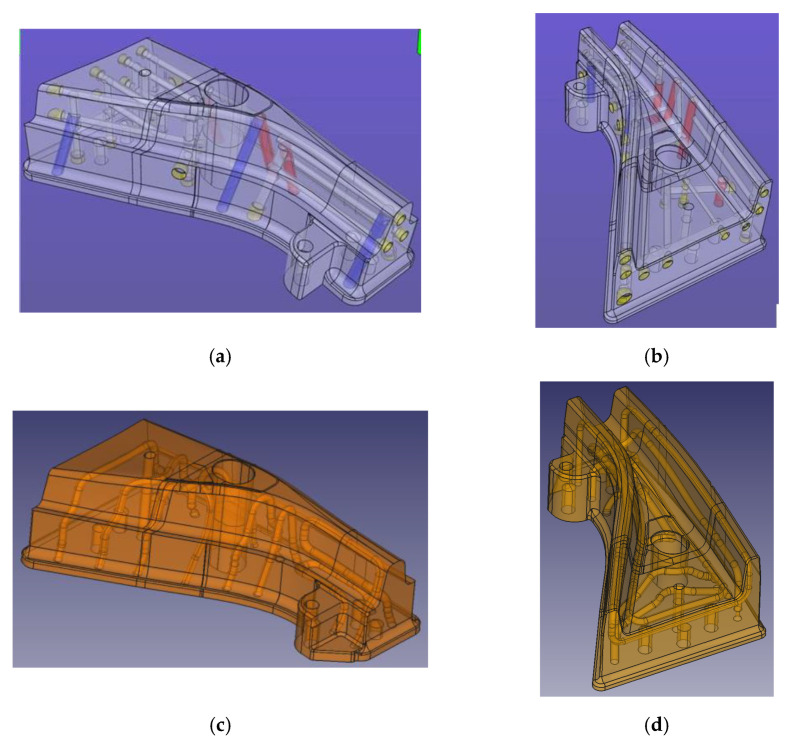
General view of the tool and cooling channel geometry; top, conventional tool: punch (**a**) and die (**b**), bottom, 3D printed tool, punch (**c**) and die (**d**).

Two copies were produced of the same tool. The first one was produced using conventional methods (machining) for both the surface and the cooling channels. This tool, produced in tool steel QRO90 and heat treated to 50–52 HRC, was identical to a die section used in an industrial component (Figure 3a,b). 

The second tool was produced by Additive Manufacturing, using the same MS1 material as in previous trials, heat treated to 52–54 HRC. The external shape and morphology were equivalent to the conventional tool. However, cooling channels were redesigned based in a conformal cooling concept, with channels following the surface and reaching zones with complicated access. This design was human-proposed as a demonstrator, no explicit FE or AI-based optimization methods were used (Figure 3c,d).

A base plate was manufactured for mounting the tools to the trial press, including fittings for blank centering and refrigeration inlets. Tests on both tools were performed using the same base plate, ensuring repeatable positioning and that any differences in performance would be due to tool design.

#### 2.3.2. Test Configuration

Tests were performed in the same press hardening line used in omega configuration tests, using the same furnace, 150 t hydraulic press and cooling equipment. 

In this case, austenitisation was performed at 930 °C and 280 s. Cycle time was 30 s, with 5 s transfer time (manual transfer), 3 s die closing time, 6 s closed die time under 35 t force, 5 s die opening.

For each experiment, a run of 20 production cycles was performed allowing tools to enter a steady state of temperature. After the 20th piece, the tool was opened, and tooling temperature measured with a thermal camera. This procedure was repeated for both punch and die, and for both conventional and Additive Manufacturing tooling sets.

**Figure 4:**
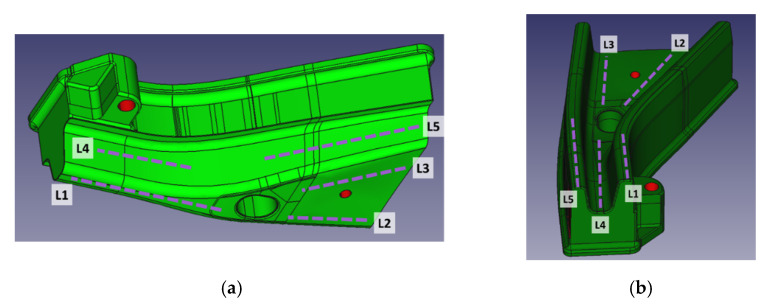
Temperature measurement zones displayed on a CAD model of the tooling: (**a**) punch; (**b**) die.

Temperature measurements were obtained with a FLIR 655SC camera, pointed at an angle of approximately 60° from the tool horizontal plane. Tool surface emissivity was calibrated using a fringe painted in a coating with known emissivity, resulting in an emissivity ε = 0.535 for the conventional die and ε = 0.69 for the additive manufactured tool; differences in emissivity were repeatable before and after production, and were thought to be related to differences in surface finish or surface oxides due to steel composition. Temperatures were measured along 5 zones in both tool and die, reporting the average temperature per zone (Figure 4).

## 3. Results

### 3.1. Wear Tests in Omega Tool Configuration

AlSi-coated boron steel is delivered with a hot dip Al-10% Si coating [1,16]. During austenitisation and heat treatment of the blanks, this coating develops a multi-layer structure formed by Al-Fe-Si intermetallic phases [16]; these phases are hard and brittle [17,18], and they are responsible for the complex phenomena in tool-sheet metal tribological interaction.

**Figure 5:**
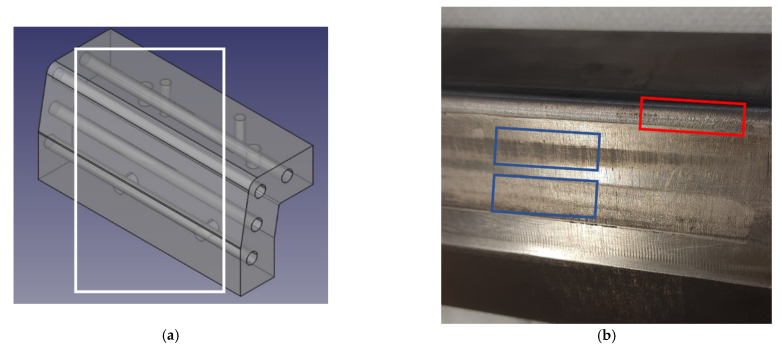
Overview of a worn lateral of the tool (3D printed side): (**a**) overview of the tool insert; (**b**) picture of the working face of a tool lateral, showing material transfer (red), dull wear fringes (blue) and the bright lateral surface where roughness measurements were taken; faint scratches can be observed in the component sliding direction.

Figure 5 shows an overview of a tool insert, corresponding to one lateral of the die. The image shown corresponds to the 3D printed insert, although both sides of the tool present a similar general aspect. A distribution of wear mechanisms was observed. Material transfer concentrated mainly in the drawing radius of the tool. Along the tool lateral, two horizontal fringes appeared where the tool lost its luster, indicating complex wear phenomena. Mild abrasion appeared in the rest of the lateral wall. Overall, this distribution of wear and damage mechanisms closely resembles that observed in production tools [17,19,20,21], and coincides with mechanisms observed in previous discussions using this same test configuration [16]. 

Based on these observations, wear on the two inserts was compared in terms of abrasive wear (observed through the modification of surface roughness), accumulation of material on the top radius, and appearance of dull fringes with combined wear mechanisms.

#### 3.1.1. Roughness Modification and Abrasive Wear

Abrasive wear in press hardening takes place due to the formation of abrasive particles flaked from the coating in the sheet metal, as well as interaction with sheet asperities [20,22,23]. Abrasive wear results in net loss of shape of the tool in the most affected zones, resulting in poor contact which is detrimental to component cooling.

**Figure 6:**
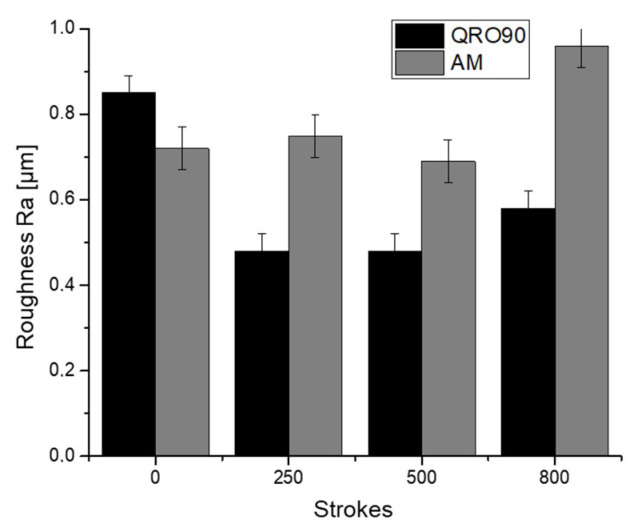
Ra roughness measurements on Surface 1 for the reference material (QRO90) and the Additive Manufacturing insert.

Abrasive wear cannot be quantified in the current study, as the 800 cycles are not enough to generate macroscopic shape deviations; however, these abrasive mechanisms can be inferred from changes in tool surface roughness particularly in zones where no material transfer is taking place, namely in the bright surface of the tool flank (Figure 5). Results are summarized in Figure 6.

Behavior of the two materials is slightly different. In the case of QRO90, there appears to be a running-in phase in which roughness rapidly diminishes in the first 250 cycles as the first contact cycles eliminate remaining tool asperities. At this point, roughness remains stable during the rest of the test, with a slight tendency to increase that could be linked to the appearance of slight scratches or minute instances of material transfer.

For the AM material, this first running-in phase is not observed; however, the material still shows an approximately stable roughness with a tendency to increase in the last inspection. Visual observation of the surfaces does not show gross scratching in either case; even though faint scratches in the component sliding direction can be identified.

#### 3.1.2. Material Transfer on Tool Radius

Accumulation of material on certain zones of a press hardening tool is one of the most typical tribological phenomena in press hardening of coated sheet steel, and has been widely described in the literature as *galling* or *adhesive wear* [2,19,20,21,22]. In this paper, as in [20], the term “material transfer” has been preferred, due to the complex mechanism leading to these features. These accumulations, often referred to as *lumps* or *transfer layers* are generated through the accumulation of dust formed from the sheet metal coating [22], which is compacted on anchoring points formed by either topological/geometrical or chemical means [17,21].

**Figure 7:**
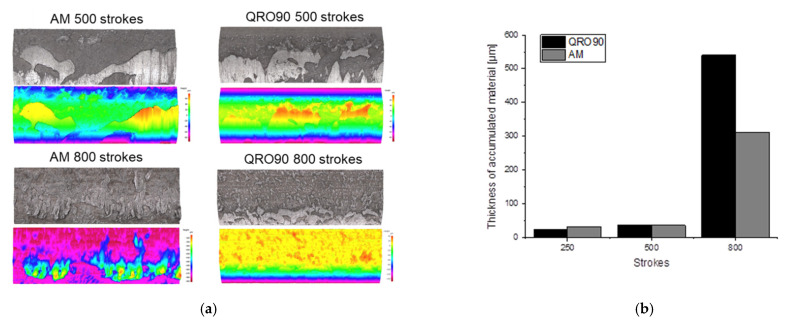
Optical topography measurements of material transfer on the tool radius: (**a**) pseudocolour and topography images; (**b**) height of the highest instance of adhered material.

Accumulation of transferred material was measured using optical profilometry on the edge of the insert radius, as shown in Figure 5. A summary of results is presented in Figure 7, showing an overview of the morphology of the adhesion features and the height of accumulated material, measured at 250, 500 and 800 strokes.

The mechanism of material accumulation is as expected for a severe change of slope over a radius, with lumps forming over the radius and coalescing into continuous layers with complex surface finish for the images obtained at 800 cycles. The thickness of these lumps evolves similarly for both materials, reaching an approximate 35 µm at 500 cycles and an almost identical severity and morphology for the two inserts. At 800 cycles, this thickness reaches very high values, over 300 µm for the AM insert and in excess of 500 µm for the conventional material. This accumulation is more severe than that reported in industrial tools [17,21,22] and is related to the severity of the test geometry. Noticeably, the QRO90 insert appears to have coalesced more completely into a continuous layer, while the 3D printed tool is still in the process of joining anchoring points. 

While this hints at a slightly better performance for the Additive Manufactured side, both tool materials appear to perform similarly, showing similar evolution over the cycles, and the significant difference in layer thickness could be related to the highly stochastic nature of the phenomenon.

#### 3.1.3. Side Wall Wear Fringes

Dull fringes, perpendicular to sheet sliding direction, were clearly observed in the lateral wall (Figure 5). These fringes were also observed in other omega-shaped setups and in industrial tools with similar geometry [17,21] and correspond approximately to radius-wall transition points at the end of the drawing stroke. Inspected by optical profilometry, these fringes show a combination of wear mechanisms, mainly material transfer in the form of small lumps combined with abrasive wear.

The width of these fringes was measured in each of the inspection intervals using a stereo microscope.

The lower fringe is generally wider, hinting at more severe pressure or contact conditions; in this case, both tools showed almost identical results.

**Figure 8:**
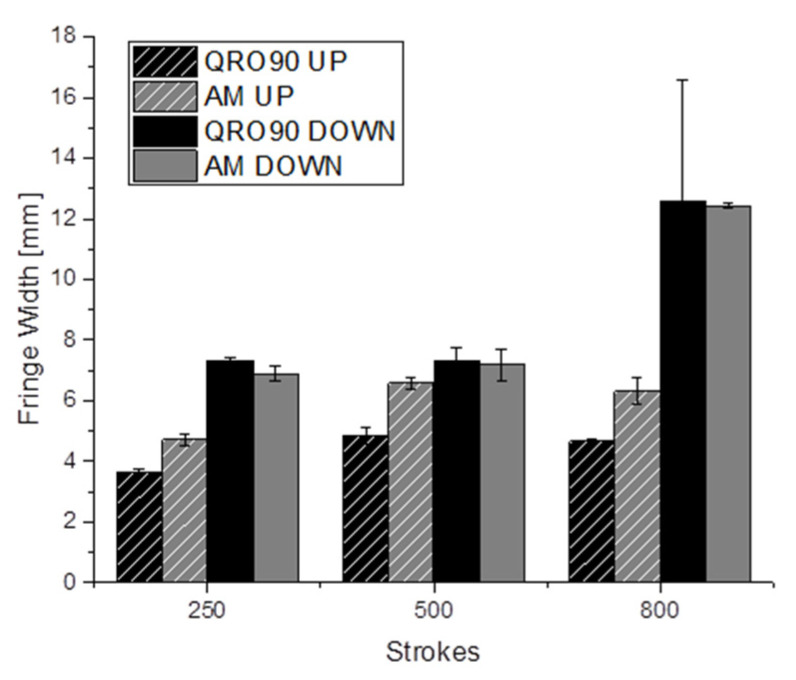
Width of the wear fringes on the tool side wall; measurements performed by stereo microscopy.

For the upper fringe, however, slight differences were observed, with the QRO90 side experiencing less severe wear and stabilizing at a lower value that the additive manufactured insert (Figure 8). 

### 3.2. Thermal Performance of Demonstrator Demo1

Four different production tests were performed, as described in Section 2.3. After 20 strokes the tools were assumed to have reached stable state, and temperature measurements were performed.

**Figure 9:**
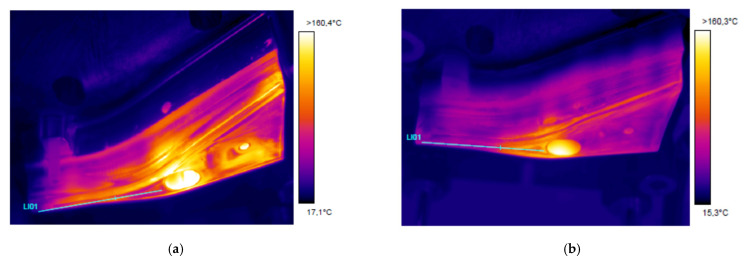
Thermal images from the punch after performing 20 production cycles: (**a**) Conventional tool; (**b**) 3D printed tool.

**Figure 10:**
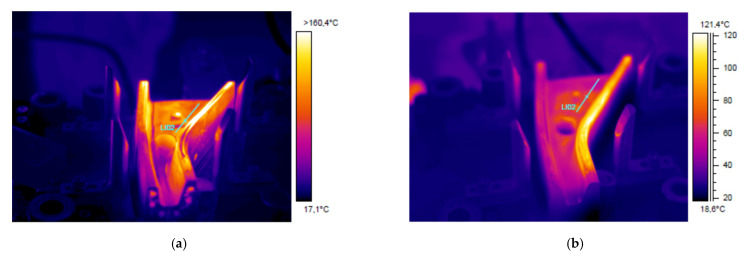
Thermal images from the die after performing 20 production cycles: (**a**) Conventional tool; (**b**) 3D printed tool. Different scales had to be used due to the differences in temperature levels and distribution.

**Figure 11:**
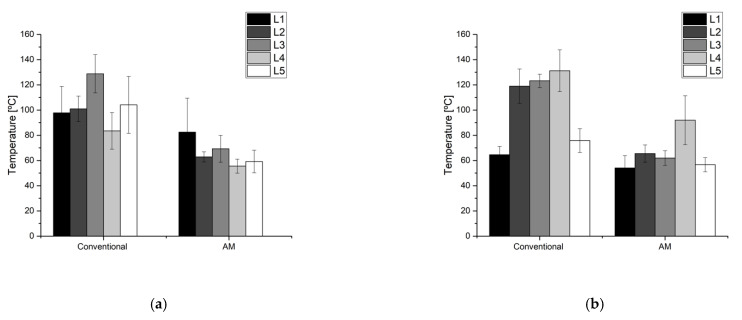
Temperatures measured in different zones of the tool, as described in Section 2.3.2: (**a**) Punch; (**b**) Die.

Thermal images qualitatively showing the temperature distribution through each of the tool parts are presented in Figure 9 and Figure 10. A plot comparing temperature in different tool areas is shown in Figure 11. In general terms, the additive manufactured tool presents overall lower temperature, as well as a more homogeneous distribution.

In both cases, the central hole shows very high temperatures. This is a measurement artifact: the geometry of the hole resulted in a locally very high emissivity, that generated an apparent high temperature point; these temperatures do not necessarily match the reality.

The conventional punch presents hot spots on the complex shape zone surrounding the central hole, on the wide zone of the upper surface and on the long lateral radius. Temperature reaches over 120 °C in these zones, contrasting with a moderate 80 °C in the less critical areas. This is due to the highly complex surface geometry, which poses severe limitations in the design of cooling channels, as it is not possible to enter bores through the tool working surface to generate a closed circuit. This forces a solution where the cooling loop is far from the surface in these points (Figure 3a).

In contrast, the 3D printed tool shows mostly homogeneous temperatures in the 60–80 °C range, with a hot spot near the central hole reaching approximately 100 °C. In this case, the free-form design of the cooling system has allowed bringing channels close to the surface even in complicated areas, resulting in much higher efficiency.

A similar temperature trend is observed for the die. The conventional die presents temperatures more than 120 °C in many areas, particularly in the wide section of the component and in the lateral “lip” dragging the sheet metal around the punch (right side wall in the images); again, these are sections where machined cooling channels find design difficulties. 

Again, the additive manufactured die showed lower and more homogeneous temperatures, in the range of 60–70 °C through the die. It must be noticed that the lateral lip is still problematic with this configuration, with the presence of a hot spot with temperatures close to 100 °C over a large area. 

Overall, the temperatures showed lower levels and less hot spots in 3D printed tools (Figure 10); however, it was not possible to completely eliminate hot spots. This is particularly true for the lateral lip discussed in the die, where the slender shape may posit refrigeration problems regardless of the design strategy employed: in this case, even free-design cooling channels may not be able to provide a full solution.

However, it is still possible that more efficient results can be reached. Tool channel design in this work was based on human design, contrasted with basic FE modelling. Based on the results of the current work, it is worth noting that the use of FEM-based or even AI-based design methodologies, such as the literature references [6,7,12] could lead to optimized solutions and to overall better cooling efficiency in very complex cases.

## 4. Conclusions

In this work, 3D printing was explored as a means to produce highly efficient press hardening dies with free-design cooling channels, using commercial AM equipment and Maraging steel as tool material. The following conclusions could be drawn:

- Additive Manufacturing appears to be a technically realistic solution for producing press hardening tools, offering a good combination of durability and performance.
- In terms of wear, 3D printed tools showed comparable performance to standard material. Wear mechanisms in both cases corresponded to those in industrial press hardening tools, and the severity of the mechanisms was overall similar in both cases, with a slight advantage for the conventional material in abrasive and combined wear mechanisms.
- Thermal performance of the 3D-printed tools was significantly better than for the conventional design. Free-design conformal cooling channels resulted in overall lower temperature and solved most of the hot spots encountered in the standard design.
- Even the design of the cooling channels was completed mainly by human design, and the performance obtained was good. However, this could potentially be improved if dedicated AI- or FEM-driven design tools were applied, as described in the literature.

## Figures and Tables

**Table 1:** Main alloying elements in the steel grades used in this work (composition in weight%).

	C	Si	Mn	Cr	Mo	Ni	Co	V
MS1	<0.03	<0.1	<0.1	<0.5	4.5–5.2	17–19	8.5–9.5	
QRO90	0.38	0.30	0.75	2.6	2.25			0.9

## Data Availability

Data is contained within the article.
